# Can chronotropic incompetence predict life-threatening ventricular arrhythmias in patients with stable ischemic heart disease?

**DOI:** 10.22088/cjim.9.2.164

**Published:** 2018

**Authors:** Yelena Rib, Gulnar Zhussupova, Gaukhar Igimbayeva, Ayan Abdrakhmanov, Seyed Farzad Jalali

**Affiliations:** 1 Astana Medical University, Astana, Kazakhstan.; 2 Department of Internal Diseases, Astana Medical University, Astana, Kazakhstan.; 3 Karaganda State Medical University, Karaganda, Kazakhstan.; 4 Depatment of Interventional Arrhythmology National Research Center for Cardiac Surgery, Astana, Kazakhstan.; 5 Social Determinant of Health Research Center, Health Resarch Institute, Babol University of Medical Sciences, Babol, Iran.; 6 Department of Cardiology, Babol University of Medical Sciences, Babol, Iran.

**Keywords:** Chronotropic incompetence, Stable ischemic heart disease, Ventricular arrhythmias, Risk factor

## Abstract

**Background:**

Chronotropic incompetence has prognostic value of all-cause and cardiovascular mortality in both patients with asymptomatic and symptomatic ischemic heart disease (IHD), regardless of traditional risk factors. The aim of this study was to investigate the relationship between chronotropic response during exercise test and the development of ventricular arrhythmias.

**Methods:**

153 patients with stable ischemic heart disease were screened and observed during the 24 months since October 2014 in a university hospital in Astana Kazakhstan. They underwent bedside electrocardiography, 24h heart rate Holter monitoring, echocardiography, exercise stress test (treadmill) for assessment of chronotropic index calculating at first contact. Holter- electrocardiography was repeated three times (at 3, 6, 12 months of follow-up period) to reveal life-threatening ventricular arrhythmias.

**Results:**

The quantity of the ventricular extrasystoles was higher in the group with low chronotropic index. Low chronotropic index increased the risk of high grade ventricular extrasystoles more than two times (P=0.015); episodes of non-sustained VT more than three times (p<0.001); and episodes of sustained VT more than nine times (p<0.001).

**Conclusions:**

Chronotropic index less than 35.6 increases the risk for life-threatening ventricular arrhythmias in patients with stable chronicle ischemic heart disease irrespectively of severe left ventricle systolic dysfunction.

According to World Health Organization data in 2012, 17.5 million people died of cardiovascular diseases as 31% of all causes of death in the world. From this number, 7.4 million people died of ischemic heart disease. Proceeding results of regional statistical reports, mortality of cardiovascular diseases in the Central Asian countries remains one of the highest in the world, making nearly 140 cases of 100 000 population. (1-3). Ischemic heart disease (IHD) is one of the most frequent reasons of mortality among cardiovascular diseases yet. Research studies for detecting the causes of mortality in patients with IHD have been conducted for more than 70 years, as a subject of a great number of studies and publications in the world. IHD has extremely wide concept from acute coronary syndrome to a hibernating chronic cardiomyocytes. According to this fact, the risks of sudden and non-sudden mortality due to different forms of IHD are various. There are known and reliable results about predictors of mortality in stable chronic IHD without severe left ventricle (LV) dysfunction (4). In predicting fatal events in patients with IHD, the huge role is assigned to exercise ECG test which opens a set of criteria for the evaluation of cardiovascular system. 

Unfortunately, the practical cardiology uses the small volume of possible tests on the basis of exercise electrocardiography (ECG) test today. This article is devoted to evaluate the prognostic value of chronotropic function of the heart. According to epidemiologic studies, the profile of changing heart rate in and after exercise test has prognostic value (5). The maximum heart rate during exercise test may have prognostic value more than duration of exercise test and ST segment depression.

In 1970’s Ellestad et al. in a retrospective study of 2700 people, who underwent exercise ECG test, showed the inability of increasing heart rate during the test which is related to a larger risk of fatal events in the next 5 years more than ischemic ST segment depression (6) and then, the term “chronotropic incompetence” (ChI) was defined. Further researches confirmed this statement, having shown that a ChI has prognostic value in both the asymptomatic and symptomatic IHD, regardless of the traditional risk factors (7, 8). According to Lauer M.S. et al. (1999), ChI is defined as the inability to achieve 85% maximum heart rate according to age, and can predict increased risk of mortality. Assessment of heart rate in recovery time after exercise ECG test is also independent prognostic information criteria. Delayed recovery of the heart rate (less than 12 beats per minute by the end of the fourth minute of the recovery period) is an independent predictor of mortality and along with Duke treadmill score, allows to predict mortality among adults (9). The aim of this study was to investigate the relationship between chronotropic response during exercise testing and the development of high grade ventricular arrhythmias (VA) in the subsequent observation.

## Methods

This is a prospective one-center cohort research conducted from October 2014 to October 2016. The research included adult patients with inclusion criteria of chronic stable IHD,with preserved (more than 50%) or mildly reduced (40-50%) LV ejection fraction (LVEF). Among the 480 patients with stable IHD, 135 of them were selected according to the inclusion criteria (108 men and 27 women) with mean age of 63 years old. All patients were hospitalized in the Cardiology Department of a university hospital in Astana, Kazakhstan. Before the beginning of the study, all patients obtained an informed consent, research did not conflict with the principles of Helsinki declaration and was approved by the local Research Ethics Committee of Astana Medical University. The exclusion criteria were: myocardial infarction, coronary artery bypass graft surgery ,percutaneous coronary intervention, acute coronary syndrome in the previous 12 months; angina pectoris class IV (Canadian Cardiovascular Society); heart failure class IV New York Heart Association (NYHA); congenital heart disease; valvular heart disease; non-ischemic cardiomyopathy; sick sinus syndrome, chronic atrial fibrillation; history of sudden cardiac death (SCD) with resuscitation; documented ventricle tachycardia (VT) and ventricle fibrillation (VF) in history; permanent administration of antiarrhythmic drugs; the presence of implanted cardiac devices, anemia, and documented thyroid diseases. 

All patients underwent anthropometric measurements, laboratory tests, 6-minutes walk test, 12-lead resting ECG, 12-lead 24-hour Holter-ECG and echocardiography was carried out on the same day as baseline examination. All participants underwent Bruce treadmill stress test protocol with the subsequent calculation of chronotropic indexes. The treadmill stress test was carried out according to guideline (12) on Shiller Intertrack RS-232 equipment. According to the obtained data, patients were divided into two groups with normal and with abnormal ChI indexes. ChI was defined as the impossibility of achievement of 85% from maximum heart rate or as a low chronotropic index (≤35,6).

**Indexes were calculated with formulas: **chronotropic index = (reached heart rate – resting heart rate) / [3/4×(220 – patient’s age) – resting heart rate] + (reached heart rate – resting heart rate) / [17.20×(220 – patient’s age) – resting heart rate]+ (reached heart rate – heart rate in the first minute of recovery period)+ (reached heart rate – heart rate in the second minute of recovery period).

The reached heart rate means maximum heart rate reached during treadmill stress test (beat per minute); and the resting heart rate means the heart rate before beginning stress test (beat per minute). Chronotropic reserve, the first minute of recovery period was calculated as a difference between the reached heart rate and heart rate at the end of the first minute of recovery period (beat per minute) (13). Some history data were refined by examining outpatient cards. In 3, 6 and 12 months from registration time in the research, all patients were invited to control visits and ambulatory 12-lead 24-hour Holter-ECG procedure. The endpoint of the research was the life-threatening ventricular arrhythmia (VA) episodes during the follow-up period. Endpoint event registration includes: episodes of life-threatening VA as episodes of non-sustained (less than 30 seconds in duration) and sustained (more than 30 seconds) ventricular tachycardia (VT), or any episodes of ventricular flutter/fibrilatioin on 12-lead 24-hour Holter-ECG (14). 

Statistical analysis: Kolmogorov-Smirnov test was used to assess the normality of distribution. The results of the quantitative variables presented as mean ± standard deviation (M±SD), and with non-Gaussian distribution as median and interquartile range (M (25, 75)). Qualitative variables are presented as absolute values ​​and percentages. Comparison of the qualitative characteristics was performed using χ² Pearson, Fisher’s exact test. Quantitative indicators are compared with each other using the student’s t-test for independent samples, and with non-Gaussian distribution using Mann-Whitney U-test. To assess the risk of life-threatening VA relative risk was calculated using analysis of contingency tables with calculation of 95% confidence interval (95% CI) by D. Katz method (15). For all types of analysis, a p<0.05 was considered statistically significant. Statistical analysis was performed using IBM SPSS statistics 20 software (IBM, USA).

## Results

Out of the 153 patients, 135 enrolled the study. Their data were completed for the analysis. Among patients, dropout occurred in 18 patients, two people died (one with sudden cardiac death, one with a stroke), and 16 people withdrew from the study of their own volition due to different reasons. Depending on the chronotropic index, patients were divided into two groups: with normal ChI (n=79) and with low ChI (n=56). 

Among the 135 patients who completed the study, there were 108 (80%) males; the average age of participants was 63 [63±6] years, with average follow-up period of 14.9±1.5 months. In table 1, the baseline characteristics of groups are indicated depending on the chronotropic index. The group with the normal chronotropic response had larger number of participants, males prevailed in both groups. There were also no differences in socio-economic status, ethnicity or the place of residency. Patients with normal chronotropic index were younger. Among patients with normal chronotropic index, about one third of them suffered diabetes mellitus type II that is nearly 1.5 times less than patients with low chronotropic index.

**Table 1:** Baseline characteristics of study groups defined by baseline chronotropic index.

**Characteristic**	**Chronotropic index >35.6** **(n=79)**	**Chronotropic index ≤35.6** **(n=56)**	**Р-value**
Male sex, n (%)	68 (86%)	48 (85%)	0.800
Average age, year	63 [57- 68]	64 [60- 69]	0.006
Native /European nationality, n (%)	65/14 (82/18%)	41/15 (73/27%)	0.244
Current smoking, n (%)	52 (65%)	39 (69%)	0.300
Walking ≥3 km a day, n (%)	36 (45%)	18 (32%)	0.054
Diabetes mellitus type II, n (%)	23 (29%)	24 (42%)	0.001
IHD history, years	9 [4.5-12.75]	7 [5.0-10.0]	0.270
Previous myocardial infarction, n (%)	32 (40%)	30 (54%)	0.020
NYHA I, n (%)	27 (34%)	8 (14%)	0.001
NYHA II, n (%)	37 (47%)	25 (45%)	0.420
NYHA III, n (%)	15 (19%)	23 (41%)	0.001
6-minute walk test, m	368±22.1	228±17.0	<0.001
BMI, kg/m²	29 [27-31]	27 [25-30]	<0.001
Resting heart rate, beats/min	75±14.8	79±12.9	<0.001
Systolic BP, mm Hg	146.8±11.7	153.5±12.6	<0.001
Diastolic BP, mm Hg	90±6.8	94±3.8	<0.001
Hemoglobin, g/dl	14.3[11.4-15.5]	13,6 [11.7-14.3]	0.038
LVDD, cm	5.2±0.90	5.6±1.18	0.002
LVMI, g/cm²	129.2±7.1	131.5±8.6	0.076
LVEF, %	54.4±7.3	49.7±3.9	0.030

BMI: Body mass index. BP: blood pressure, LVDD: Left ventricular diastolic diameter, LVMI: Left ventricular mass index.

**Figure 1:**
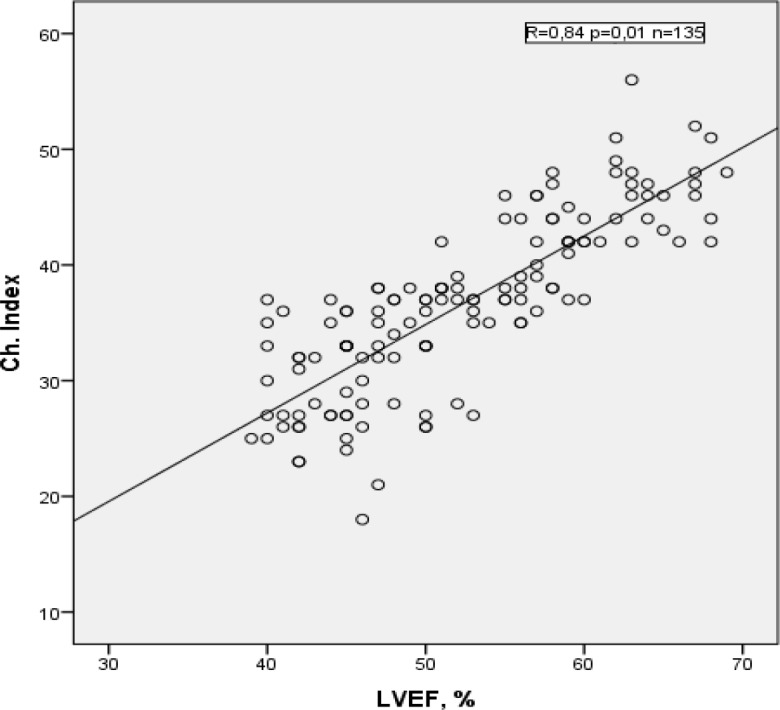
Correlation between chronotropic index and LVEF in patients with stable IHD without severe LV dysfunction.

As findings in table 1, there was lack of relationship between groups with normal and low chronotropic index. On the other hand; sex, average age, nationality, smoking and even degree of acceptable load (proceeding daily walking) did not differ in both groups. But the presence of type II diabetes mellitus and the previous myocardial infarction in group with abnormal chronotropic response considerably exceeded comparing patients with normal heart rate response during stress test.Pathological chronotropic index prevailed also among patients with symptomatic heart failure. Patients with NYHA I heart failure were more in group with normal chronotropic response whereas NYHA III – in group with low index. There was approximately the same number of patients with NYHA II heart failure in both groups. With Spearman’s rank test, we revealed direct strong correlation between chronotropic index and LVEF (P=0.84, P=0.01, n=135). Moderate decrease of LVEF correlated with low chronotropic index (figure 1).

Average blood pressure measured in hospital was higher in group of participants with low chronotropic index. The average resting heart rate in patients with abnormal ChI exceeded comparing to persons with normal chronotropic index. The analysis of transthoracic echocardiographic parameters confirmed the relationship between increased dimensions and decreased global contractility of left ventricle in patients with low chronotropic response, but LV myocardial mass index did not differ significantly in both groups. Baseline electrocardiographic abnormalities depending on chronotropic index were distributed as follows: LV hypertrophy on ECG was revealed in 52 (65%) patients in group with normal chronotropic index compared with 39 (69%) patients in group with low index (P=0.36). The average night heart rate on 24-hour Holter-ECG was not different; 64±9.8 beats/min in group with normal chronotropic index and 66±7.2 beats/min in patients with low chronotropic index (P=0.08). Significant differences in two groups were revealed only in average daily heart rate during 24-hour Holter-ECG; the average daily heart rate was 74±4.8 beats/min in group with normal chronotropic index, and 83±7.6 beats/min in group with low chronotropic index (P=0.02).

Results of treadmill exercise stress tests were coordinated with data of several studies (16). Patients with low chronotropic index reached smaller METs during the test and had more rigid profile of heart rate during recovery period (table 2). The common quantity of the ventricular extrasystoles (VE) on Holter-ECG was higher in the group with abnormal chronotropic index. The low chronotropic index in patients with stable IHD increases the risk of high grade VE development more than two times; for episodes of non-sustained VT, exceeding three times and for episodes of sustained VT, nine times more (table 3).

**Table 2:** Exercise ECG test features depending on the chronotropic index.

**Characteristic**	**Chronotropic index >35.6** **(n=79)**	**Chronotropic index ≤35.6** **(n=56)**	**Р-value**
Achieved METs	8.7±3.8	6.0±2.7	0.002
Percentage of peak heart rate	80 [69-94]	65 [56-77]	<0.001
Peak heart rate	148 [135-167]	118 [98-138]	<0.001
Heart rate in the end of the first minute of recovery period	121 [106-140]	110[103-128]	<0.001
Chronotropic reserve in the first minute of recovery period	17 [12-29]	9 [6-16]	<0.001
Mean chronotropic index	46.8 [38.3-59.4]	26.7[18.9-32.6]	0.01

METs = metabolic equivalence

**Table 3:** Relative risk of ventricular arrhythmias defined by chronotropic index (according to Holter-ECG data)

**Characteristic**	**Chronotropic index >35.6** **(n=79)**	**Chronotropic index ≤35.6** **(n=56)**	**RR** **(95% CI)**	**Р-value**
VE, n/24 hours	189 [136- 248]	234 [142- 364]	3.1(1.80-4.70)	0.030
VE III-IV Lown-Wolf grading, n (%)	18 (22)	28 (50)	2.30(1.22-2.76)	0.015
Non-sustained VT on Holter-ECG, n (%)	8 (10)	13 (23)	3.18(1.38-7.33)	<0.001
Sustained VT on Holter-ECG, n (%)	1 (1.2)	4 (7)	9.19(2.08-34.7)	<0.001

## Discussion

Detection of** a**rrhythmogenesis markers is important for the prevention of SCD in IHD patients. In practice, primary prevention of SCD for patients with IHD without previous life-threatening VA mainly focuses on LVEF. Previous studies also indicated a significant role of chronotropic function of heart rate in the prediction of sudden cardiac death. In the present research, the possibility of chronotropic response indexes and possible predictors of fatal arrhythmia among patients with IHD is considered. Among the 56 patients with low chronotropic index, the relationship with development of life-threatening VA was revealed. The fatal coronary events and increased cardiac mortality recorded in patients with low chronotropic response (17) can have the common pathophysiological mechanisms with fatal arrhythmia in this population.

The most common and the studied mechanism is sympathetic dysfunction in chronic IHD involving myocardium and conductive system of the heart. One of the pathophysiological conditions may be post-synaptic desensitization of the beta-adrenergic receptor pathway in the sinoatrial node. Frequent activation of sympathetic nerves will cause the downregulation of beta-adrenergic receptors, leading to post-synaptic desensitization (18, 19). Perhaps, this explains the fact of a high resting heart rate in patients with low chronotropic index but non-achievement submaximal heart rate during an exercise test. Changing the balance between sympathetic and parasympathetic cardiac rhythm regulation as one of the arrhythmogenicity markers, has been proven in numerous researches of rhythm variability in chronic ischemia.

Lauer et al. reported a similar result that ChI was associated with smoking in a health population-based cohort study. The mechanism linking smoking to ChI remains unclear, but there may also underlie the postsynaptic desensitization of the beta-adrenergic receptor pathway (20). Frequent activation of sympathetic nerves provoked by daily smoking may deteriorate post-synaptic sensitivity of the beta-adrenergic receptor pathway, possibly leading to ChI during exercise testing. However in the present research, the number of smoker patients in both groups significantly did not differ; and relationship between duration of smoking and number of smoked cigarettes in a day was not found out.

In addition, ChI has been reported to be associated with left ventricular dilation and hypertrophy (21). The LVMI in both groups did not differ considerably, whereas a dilatation of LV cavity prevailed in patients with low ChI. Considering that reduced LVEF plays a significant role in increasing of all-cause (non-arrhytmic and arrhythmic mortality), it is also possible to assume the existence of communication between reduced LVEF and impaired chronotropic response. This fact was legibly elicited in the present research and confirmed in the previous researches (22). 

An opportunity of measuring chronotropic index as a marker of electrical instability of myocardium is extremely tempting in view of simplicity of inspection and calculation. It may be possible to use it both in screening programs and in the evaluation of patients for primary prevention of SCD.

**Study limitations**: The research was not randomized with small sample size and short duration of the follow-up period. Some of the data used to determine the risk factors (such as family history of coronary heart disease, SCD, alcohol drinker, regular physical activity) are subjective and anamnestic without the possibility of objective verification. The present study did not include a stress test using pharmacological autonomic nerve blockades. The ECG and Holter-ECG were performed during patient admission in the hospital on drug-free background after at least two days of drug withdrawal on ambulatory basis therapy. A small number of patients with comorbidities were prescribed drugs in the follow-up period, indirectly affecting the electrophysiological properties of the myocardium including macrolide antibiotics and short-acting bronchodilators, which could be the cause of changes in the ECG and Holter-ECG. 

In conclusion, chronotropic index less than 35.6 increases the risk for life-threatening VA development in patients with chronic ischemic heart disease. It requires longer observational studies for better evaluation of clinical outcomes with precise study on chronotropic function during ECG stress test as the possible predictor of fatal arrhythmia.
